# Exploring the experiences of substitute decision-makers with an exception to consent in a paediatric resuscitation randomised controlled trial: study protocol for a qualitative research study

**DOI:** 10.1136/bmjopen-2016-012931

**Published:** 2016-09-13

**Authors:** Melissa J Parker, Sonya de Laat, Lisa Schwartz

**Affiliations:** 1 Division of Pediatric Critical Care, Department of Pediatrics, McMaster Children's Hospital and McMaster University, Hamilton, Ontario, Canada; 2 Department of Clinical Epidemiology and Biostatistics, McMaster University, Hamilton, Ontario, Canada; 3 Division of Emergency Medicine, Department of Pediatrics, The Hospital for Sick Children, and University of Toronto, Toronto, Ontario, Canada; 4 Department of Philosophy, McMaster University, Hamilton, Ontario, Canada

**Keywords:** Consent, QUALITATIVE RESEARCH, ETHICS (see Medical Ethics), ACCIDENT & EMERGENCY MEDICINE, INTENSIVE & CRITICAL CARE

## Abstract

**Introduction:**

Prospective informed consent is required for most research involving human participants; however, this is impracticable under some circumstances. The Tri-Council Policy Statement: Ethical Conduct for Research Involving Humans (TCPS) outlines the requirements for research involving human participants in Canada. The need for an exception to consent (deferred consent) is recognised and endorsed in the TCPS for research in individual medical emergencies; however, little is known about substitute decision-maker (SDM) experiences. A paediatric resuscitation trial (SQUEEZE) (NCT01973907) using an exception to consent process began enrolling at McMaster Children's Hospital in January 2014. This qualitative research study aims to generate new knowledge on SDM experiences with the exception to consent process as implemented in a randomised controlled trial.

**Methods and analysis:**

The SDMs of children enrolled into the SQUEEZE pilot trial will be the sampling frame from which ethics study participants will be derived. *Design:* Qualitative research study involving individual interviews and grounded theory methodology. *Participants:* SDMs for children enrolled into the SQUEEZE pilot trial. *Sample size:* Up to 25 SDMs. *Qualitative methodology:* SDMs will be invited to participate in the qualitative ethics study. Interviews with consenting SDMs will be conducted in person or by telephone, taped and professionally transcribed. Participants will be encouraged to elaborate on their experience of being asked to consent after the fact and how this process occurred. *Analysis:* Data gathering and analysis will be undertaken simultaneously. The investigators will collaborate in developing the coding scheme, and data will be coded using NVivo. Emerging themes will be identified.

**Ethics and dissemination:**

This research represents a rare opportunity to interview parents/guardians of critically ill children enrolled into a resuscitation trial without their knowledge or prior consent. Findings will inform implementation of the exception to consent process in the planned definitive SQUEEZE trial and support development of evidence-based ethics guidelines.

**Strengths and limitations of this study**

- Rigorous qualitative methodology that aligns with CORE-Q requirements.
- Protocol carefully constructed to prospectively manage any real or perceived competing interests of the principal investigator of the parent trial on the dependent ethics study.
- Novel safety plan protocolises procedures for management of any obvious emotional distress in qualitative research participants witnessed by research staff during or at completion of the interview process.
- Qualitative data will be based on parent/guardian recall, which may be incomplete and/or inaccurate.

## Background

### Consent and health research ethics

Informed consent is a key requirement for the ethical enrolment of human participants in biomedical research.^1^ The principle of Respect for Persons involves recognising the autonomy of all individuals, and protecting those who are vulnerable and unable to exert their autonomy.^2^ Under the principle of Autonomy, human beings have the right to self-determination. Supporting valid informed consent involves providing potential participants information about the purpose of the research, explaining the relative risks and benefits, describing the nature and duration of participation required and explaining the voluntariness of participation, all within a context free from coercion. Where an individual is incapable of providing informed consent, it has become an accepted practice for a substitute decision-maker (SDM) to act on their behalf for decision-making related to research participation, though SDM decisions do not necessarily accurately represent participant wishes.^3–5^

Other principles underpinning the ethical conduct of research in human participants include Concern for Welfare and Justice.^2^ Researchers must protect and promote the welfare of participants and minimise the risks of research participation, while SDMs must act according to the patient's expressed wishes, or if these are not known they should act to promote an incapable person's best interests. Justice in the research context is often characterised as fairness such as fair practices of recruitment, design, etc, and fair distributions of benefits and burdens of research. Individuals must have the opportunity to participate.^6^ This is challenging when research participation may be effectively denied where a rigid requirement for prospective informed consent is imposed. Such requirements effectively halt research for individual medical emergencies, where prospective informed consent is impracticable.^7^ Even for research occurring outside of the acute care context, it has been argued that fully informed consent is an ideal that is difficult, if not impossible, to achieve.^8^ ^9^

More is being learnt about how valid informed consent can be evaluated and ensured by a researcher.^10–12^ In the clinical and research context, individuals often rely on the advice of family and others when making decisions. Many individuals in fact do not want to weigh different choices or carry the burden of making such a judgement themselves.^13^ Recent research involving parents of children with cancer has explored parental decision-making^14^ as well as comprehension and satisfaction with informed consent,^15^ revealing that trust and confidence in the medical team rather than comprehension of study details inspires participation.^14^ ^15^

### Departures from the general principles of consent and the Tri-Council Policy Statement 2: Ethical Conduct for Research Involving Humans

In many jurisdictions, including Canada, it is recognised that departures from the general principles of consent are necessary to enable human research for certain conditions, supporting a utilitarian point of view. This provides a mechanism to support research in areas that would otherwise not benefit from the rigorous evaluation required to advance medical science. The Tri-Council Policy Statement (TCPS) outlines two circumstances under which departures from the general principles of consent can be ethically supported.^16^ These include (1) altered consent in minimal risk research (Article 3.7) and (2) exception to consent for research in individual medical emergencies (Article 3.8).

### Exception to (prospective informed) consent

Exception to consent for research in individual medical emergencies can be considered as the enrolment of a participant into a study without their prospective informed consent or that of their SDM where the individual is incapable and where no prior directive is known to exist. Implementing an exception to consent process (sometimes referred to as deferred consent) involves several key elements that must be meticulously addressed during study planning: (1) procedures for participant enrolment, (2) subsequent notification of the participant or SDM of their enrolment in a research study, (3) approach by a member of the research team for full discussion about the study and request for consideration for consent (and assent as applicable) for continued participation and (4) management of circumstances where consent for ongoing participation is declined. Depending on the jurisdiction in which the research is conducted, regulations vary and community consultation and/or notification may be a requirement.^17^ While resuscitation research following community consultation is possible,^18^ this does increase costs and raises further questions regarding what is meant by community, who should be consulted, how and to what degree. It has been argued that such requirements effectively serve as a barrier to conducting resuscitation research.^19^ ^20^ Community consultations also do not ensure that enrolment is acceptable at the individual level.

Importantly, little research has been conducted on the direct experience of SDMs with the exception to consent process. Interviews with capable adult survivors of critical illness reveal that only a minority finds enrolment followed by deferred consent unacceptable for research in medical emergencies.^21–23^ From a paediatric perspective, a recent systematic review evaluating the use of alternative consent models in research for life-threating paediatric emergencies found just 11 articles, with none specifically evaluating the experience of SDMs with deferred consent.^24^ Work to date has focused on community consultation in light of US regulations,^25–30^ feasibility evaluation for hypothetical studies^31^ ^32^ and interviews with parents of critically ill children.^33^ ^34^ A survey study of UK parents whose children had suffered from bacterial meningitis or meningococcemia within the past 5 years found that deferred consent was acceptable to the majority of respondents, though the views of bereaved versus non-bereaved parents differed to some extent.^33^ In contrast, parents who had received tertiary obstetric or neonatal care expressed low support for neonatal resuscitation research involving the use of a waiver, deferred or opt out consent model, though these authors noted the limitations of their work and expressed a need for more rigorous research involving parental interviews.^34^

### Context for the proposed research: an opportunity to evaluate SDM experiences

MJP is the principal investigator for the SQUEEZE pilot randomised controlled trial (RCT) (NCT01973907).^35^ This pragmatic, two-arm, parallel group, open-label pilot RCT opened recruitment at McMaster Children's Hospital (MCH) in January 2014. The overall objective of SQUEEZE is to determine in children with septic shock whether resuscitation involving less intravenous fluid and more medications (fluid sparing) compared to aggressive intravenous fluid (usual care) results in improved clinical outcomes without an increased risk of adverse events.^35^ Management in the fluid-sparing (intervention) and usual care (control) arms fall within the broad scope of current treatment guidelines.^36^ ^37^ SQUEEZE was inspired by recent evidence suggesting that too much intravenous fluid may lead to worse outcomes for patients with septic shock, including serious complications or even death.^38–40^ However, a strategy that uses less intravenous fluid and more medications may also have important adverse effects. Current septic shock resuscitation guidelines^36^ ^37^ do not specify how much intravenous fluid is optimal, generating significant debate on this issue.^41–45^ A high-quality RCT is therefore required to define optimal resuscitation care.

The primary objective of the SQUEEZE pilot trial is to determine the feasibility of a definitive multicentre RCT.^35^ Approval to conduct this pilot RCT was granted by the Hamilton Integrated Research Ethics Board (HIREB), including approval for the use of an exception to consent process, without which the RCT would not be feasible. SQUEEZE provides an opportunity to generate new knowledge on parental experiences with an exception to consent.

#### Primary objective

The primary objective is to conduct a study exploring the experiences of SDMs with the ‘exception to consent’ process in the context of a pilot RCT comparing two resuscitation strategies for paediatric septic shock.

### Research questions

1. What is the experience for parents and guardians asked for consent for their child to remain in a study/RCT after the main intervention has taken place?
  - concerns;
  - good experiences;
  - preferred practices.
2. Why do parents/guardians choose to continue or withdraw their child from ongoing participation?
  - reasons
    ethical frames of reference used
  - emotional responses
    ethical distress
3. What strategies, responses, resources, etc, employed by consenters to recruit families did SDMs feel helped them make their decisions? What could have been improved?

## Methods/design

The nature of the topic under consideration, and the exploratory aspect of the study, calls for a qualitative research approach. Qualitative research is particularly appropriate where relevant variables have not been identified^46^—a criterion especially salient to this topic area, where so little research has been performed. As well, experiences identified as ethically difficult will likely rely upon social norms about, for instance, the value of patient autonomy and other ethical constructs. Qualitative research is well suited for unpacking tacit assumptions.^46^ Methods include focus groups and individual interviews. Because of the sensitive nature of the participant, we have chosen individual interviews for this study. *Study design:* Qualitative research study involving individual interviews and grounded theory methodology. *Study setting:* MCH.

The sampling frame will include parents/guardians approached for consent for their child to remain in the SQUEEZE pilot trial. All parents/guardians of children enrolled into this RCT will be invited to take part in the ethics study, including parents who refuse continued involvement in order to capture reasons for consent and refusal. Even parents/guardians of SQUEEZE participants who do not survive their illness will be invited to participate as it is important to understand their experiences, which may differ from parents/guardians of septic shock survivors.^33^

The optimal timing for individual interviews for qualitative research in relation to a specific event has not been described in the literature. For this study, we seek to balance a desire for proximity with the need to be family centred in arranging a suitable interview time, recognising and respecting that the most appropriate time will differ among the participants—who may include grieving parents. We anticipate that most parents/guardians will be interviewed by the Qualitative Research Assistant (QRA) within 1 month of their consent encounter. Two parents/guardians for a given SQUEEZE participant may be interviewed for the ethics study as individual participants.

### Eligibility criteria

#### Inclusion criteria

1. Parents/guardians approached for consent for their child to remain in the SQUEEZE pilot trial.
2. Where two parents/guardians are involved in decision-making related to the consent process, both parents/guardians are eligible for inclusion in the qualitative ethics study.

#### Exclusion criteria

1. Parents/guardians who have indicated on the SQUEEZE pilot trial consent form that they do not wish to be approached for this qualitative study.

### Screening, recruitment and accrual of participants

Screening for eligible participants for the qualitative ethics study will be performed by the SQUEEZE pilot trial Clinical Research Assistant (CRA). The SQUEEZE pilot trial CRA will inform the QRA once a potential ethics study participant is identified. The QRA will be provided with the minimum necessary contact information required to approach the potential ethics study participant. The QRA will also be advised of the SQUEEZE pilot trial participant's last known vital status, so that the QRA will know if they are contacting a bereaved parent/guardian.

The QRA will approach parents for participation in the qualitative ethics study if:

1. The parent(s)/guardian(s) indicate agreement to being approached for the qualitative ethics study on the SQUEEZE pilot trial consent form. OR
2. The parent(s)/guardian(s) decline consent for continued participation of their child in SQUEEZE.*Rationale:* We anticipate that parent/guardian refusals for continued participation in SQUEEZE may involve rejection of the consent form in its entirety. We believe that it is very important to provide an opportunity for all parents/guardians to share their views regarding the deferred consent process in the qualitative study, and not having an opportunity to approach these parents/guardians risks sample bias. OR
3. Parent(s)/guardian(s) of children enrolled into SQUEEZE prior to implementation of the revised consent form which requests permission to approach for the ethics study.

The QRA will approach eligible parents/guardians for consent for participation in the ethics study given that they are not affiliated with the SQUEEZE pilot trial and therefore not in a position that could be perceived as a potential conflict of interest. Parents/guardians will be approached at least one calendar day after they have provided or declined consent for continued participation in SQUEEZE, and at a time when their child is either medically stable or no longer in the intervention phase of the SQUEEZE trial. The QRA will communicate regularly with the SQUEEZE pilot trial CRA to determine the earliest appropriate time to approach eligible parents/guardians regarding participation in the ethics study.

Given the severity of illness of children with septic shock, we expect that most parents/guardians will be invited in person, but where not possible we will contact them by telephone or if required by letter mail (see online supplementary files S1 and S2 for contact transcripts). Eligible parents/guardians will be informed of the nature of the current ethics study and asked if they would be willing to participate in an interview about their experience with the exception to consent process. All parents, even those who decline continued participation in SQUEEZE, will be provided the information sheet for the ethics study and advised that this is a separate study in which they are under no obligation to participate.

10.1136/bmjopen-2016-012931.supp1 Supplementary File 1: Telephone Contact Transcript: Invitation to Participate in Study

10.1136/bmjopen-2016-012931.supp2 Supplementary File 2: Letter mail Contact Transcript: Invitation to Participate in Study

Individuals who indicate an interest in participating in the ethics study will be provided with a copy of the study information and consent sheet that has been reviewed with them. For most potential participants, the study invitation will occur in person while their child is still admitted to hospital. For parents/guardians whose children are no longer in hospital, the study invitation will occur by telephone or by letter mail where attempted telephone contact is unsuccessful. Interested participants will be sent a formal information and consent sheet mailed with a postage-paid return envelope, and they will be asked to sign and return it once they are satisfied that any questions have been answered. An interview will then be scheduled.

### Study procedures

Semi-structured interviews will be conducted on the basis of open-ended questions that define the domain of inquiry, from which the interviewer or interviewee may diverge in order to pursue an idea or respond in more detail.^47^ Part of the aim of qualitative interviews is to discern the interviewee's own framework of meanings.^48^ Interviewers will be trained to avoid imposing their own understandings of, for instance, what constitutes an ethical issue. Participants will be encouraged to elaborate, in depth and detail, on their experience of being asked to consent after the fact. In particular, participants will be encouraged to describe what went on in the consent process and how they responded.

We expect that many of the qualitative interviews will be able to be conducted face-to-face, although some will need to take place by telephone. Decisions regarding on-site versus telephone interviews will be guided by our discussions with the study participants, based on their availability and preference. Several suitable interview rooms are available at MCH. Interviews may also be conducted at the Communications Resource Laboratory, which is located on the McMaster University campus immediately adjacent to MCH. Interviews will be taped and professionally transcribed by a transcriptionist who has signed a confidentiality agreement.

Anticipating that necessary comfort and rapport will not always be achieved in a single interview, and in order to allow an opportunity to explore emerging analyses with study participants (see data analysis), we plan second interviews for approximately one-third of the sample. We expect that a higher proportion of second interviews will occur by telephone; however, where possible, these will also occur face-to-face. In such cases, we will attempt to time interview appointments with any planned hospital follow-up clinic visits or otherwise defray the costs of travel to the hospital for the participant.

Some research participants will have had very difficult experiences related to their child's illness. We will hire an interviewer with skill and experience in responding to trauma, and have information at hand about resources for support beyond the interview. ‘Process consenting’^49^—ongoing assessments of the participants' willingness to continue with the interview—will also be undertaken. Participants will be reminded that they can withdraw at any time and can ask to have the recorder turned off or not respond to specific questions, and this will not affect their/their child's care.

### Blinding

The QRA and the Bioethicist (LS) will have no knowledge of whether the child of the parent/guardian enrolled into the ethics study was assigned to the treatment or control arm in the RCT. As the SQUEEZE pilot trial is open label, parents/guardians may recall and divulge this information during the interview process. There are no planned analyses according to SQUEEZE intervention assignment. Procedures for unblinding are not applicable.

### Study outcomes

Themes will be identified during the iterative data review process.

### Sample size

We anticipate that interviews with 25 participants will allow for categorical saturation. While this is generally the point at which no new or relevant data are emerging regarding core categories, and the categories are well developed in terms of properties and dimensions, we understand that saturation is largely an ideal to which we aim; however, our goal is more towards gathering a breadth of experiences to learn from.^50^ We are prepared to interview more to accommodate category development.

### Participant timeline

We expect that ethics study participants will be in contact with research team over a period of on average 1 month, from the time consent is obtained, until the interview process is completed.

### Data collection methods

The qualitative interviews with eligible and consenting parents/guardians will be conducted one-on-one with the QRA, ideally in person but where not possible these will be conducted by telephone. Where families are located or reside within the greater Hamilton area (no more than 50 km from McMaster University), and if it is the preference of the family, interviews can be conducted at a location of the participants' choice. When an interview is to be conducted off site, the QRA will notify one of the study investigators of the interview time and location, and confirm her safe departure once the interview has been completed. If at any time during the interview process the QRA is concerned for her safety, the interview will be discontinued and she will exit the premises. The QRA will carry on her person a cellular telephone at all times to facilitate urgent communication if required. The interviews will follow the attached interview guide (see online supplementary file S3). Interviews will be recorded on a digital medium (digital voice recorder), after which point the interviews will be transcribed. The interview transcripts constitute the qualitative data.

10.1136/bmjopen-2016-012931.supp3 Supplementary File 3: Qualitative Interview Guide for Study

Other data of contextual relevance to this study will be shared between the SQUEEZE Pilot Trial Research Team and the Qualitative Ethics Study Team as approved by our Research Ethics Board (REB). These data are collected either as described in the SQUEEZE pilot trial protocol or, in the case of records regarding operationalisation of the deferred consent approach, supporting documentation as recommended in the TCPS. An example of the former would be the highest PELOD2 score (a validated score indicating the severity of organ dysfunction in the participant),^51^ whereas an example of the latter would be documentation of decision-making of the research team regarding when to approach SDMs for full informed consent. Data that MJP, as principal investigator for the SQUEEZE pilot trial, proposes to share with the Qualitative Ethics Study Team are detailed in online supplementary file S4.

10.1136/bmjopen-2016-012931.supp4 Supplementary File 4: Data the PI of the SQUEEZE Pilot Trial requests to share with members of the linked Qualitative Ethics Study Research Team

### Data management

Data will be anonymised at the time of transcription. Personal names, locations and dates will be removed. All anonymised data will be stored on a password-protected computer in a locked office. Paper data with identifying markers intact (eg, signed consent forms) will be kept in a locked cabinet in a locked institutional office. Electronic data with identifying markers intact (eg, contact information, code key) will be kept in a locked file, on a password-protected computer in a locked institutional office. Audio recordings will be stored in a password-protected file on a password-protected computer in a locked office and stored securely at McMaster University for 10 years.

### Plans for collection and use of personal health information

Collection of participant identifiers will be limited to those determined to be necessary for study purposes. Participants will be assigned a unique letter/number identifying code, with participant identifiers kept separate from other data collected. The file linking participant code numbers to identifying information will be maintained in a locked electronic file on the password-protected computer of the QRA.

### Qualitative analyses

Data gathering and analysis will be undertaken simultaneously so that emerging propositions can inform and be tested in subsequent steps of the research process^52^—for instance, tentative generalisations will be shared with research participants in second interviews; they will suggest alternative interpretations for us to consider, or confirm our directions.

The investigators will collaborate to develop a preliminary coding scheme, and data will be coded using the qualitative software program NVivo (NVivo for Mac, Version 11).^53^ Constant comparison^54^—comparing coded data within a single transcript and across transcripts—allows us to explore the relevance of concepts and refine them in dialogue with the data, and begin to formulate new concepts related to the phenomenon of interest. An ongoing commitment to seek alternative explanations for phenomena, and to identify and account for negative instances, helps secure the quality of the analysis.^55^

While sensitising concepts assist with the process of data reduction and the development of initial codes, the inductive nature of qualitative research means that core concepts often only emerge during the investigation.^48^ The coding scheme will be revised towards the midpoint of the study to reflect emerging concepts. All investigators will review selected excerpts of data to check the category assignment, to ensure a reliable and valid foundation for analysis.^56^ The new coding framework will be applied to the data. In the final stage of analysis, the properties of core concepts will be fully elaborated^48^ and related to existing literature.

Obviously, given the anticipated sample size, we do not intend to represent the population in any statistical sense. Our intent is rather to generate concepts^57^—concepts about the experiences of parents/guardians who are asked to provide consent after being informed that their child has already undergone randomisation and received the study intervention. The extent of the grounds for relevance depends in part on the similarities of the participants and their experiences to the population and situations to which application is sought.^58^ One of our tasks will be to carefully describe the characteristics of participants, and the situations in which they were asked for consent for the SQUEEZE trial such that users of the research can assess the applicability of concepts generated by this study to their own situations.

### Other analyses

We do plan to present descriptive data regarding the subpopulation of children whose parents/guardians participated in the qualitative ethics study as well as the flow of the exception to consent process for these children and their SDMs. This will provide important context for readers in which to interpret our qualitative findings. These data will be summarised using descriptive summary measures: expressed as mean (SD) or median and IQR as appropriate for continuous variables and count (per cent) for categorical variables. Statistical analyses will be performed using SAS V.9.2 (SAS Institute, Cary, North Carolina, USA).

### Analysis population and plans for handling of missing data

The analysis population will consist of eligible parents/guardians who participate in the qualitative interview process. Specific strategies are not required for missing data in qualitative research studies.

### Potential harms and ancillary support

While unlikely, breach of participant privacy and/or confidentiality is always a possibility in research. We will mitigate these risks through our procedures for storage of study data and documents and ensuing compliance with applicable laws and regulations. A less obvious potential risk of study participation is the potential for the qualitative interview process to trigger emotional distress in research participants through recall of experiences while their child was critically ill. Recognising this potential for retraumatisation, our protocol includes procedures for addressing any obvious emotional distress directly observed by the QRA during or at completion of the interview process (see online supplementary file S5).

10.1136/bmjopen-2016-012931.supp5 Supplementary File 5: Procedures for Responding to Obvious Emotional Distress in a Qualitative Research Participant

### Study monitoring and audits

The Steering Committee (LS and MJP) will monitor study progress and report any adverse events to the HIREB. A Data Safety and Monitoring Board is not required given the qualitative study design. There are no planned audits for this study. This study may be subjected to audit by the HIREB or by the granting agencies supporting this work.

### Ethics

The HIREB has provided approval to proceed with this study (HIREB Project 14-807). Eligible parents/guardians will be approached by the QRA for full informed consent (see study procedures). Any modifications to study procedures must be communicated to, reviewed by and approved by the HIREB through a formal amendment request. The principal investigators will be responsible for communicating approved changes in the study protocol to research staff.

### Declaration of financial and other competing interests

MJP is principal investigator for the parent study, ‘Pilot Study for the SQUEEZE Trial’. We have carefully structured our protocol and research plans to mitigate any real or perceived impact of this potential competing interest on the integrity of the dependent ethics study. Such measures include LS serving as the principal investigator responsible for study oversight, including REB correspondence, housing and security of the qualitative data and oversight of the QRA. MJP will not be involved in conduct of the qualitative interviews or have access to primary/source data, for example, audio recordings or transcripts.

### Communication of study results

We will present our findings at national/international scientific meetings and publish these as a manuscript in a full open-access peer-reviewed journal. There are no publication restrictions or data sharing arrangements for this study.

### Authorship eligibility

Authorship will be determined in accordance with recommendations of the International Congress Medical Journal Editors.^59^ Medical/professional writers will not be involved in manuscript preparation.

### Plans for public access to the full protocol

We are publishing this study protocol to promote our work and to make this accessible to interested parties.

### Qualitative data access

Anyone may contact the investigators to request access to the aggregate qualitative data generated by this study. Transcripts, with all identifying information removed, will be archived and made available to other researchers via Scholars Portal Dataserve network, though McMaster University (http://library.mcmaster.ca/rdm/preserving/archiving.) Access to the full data set and/or complete transcripts may require contacting the study participants to request consent related to the specific use proposed. No one other than LS and her research team, all of whom have signed a confidentiality agreement, will have access to the audio recordings of the interviews.

## Discussion

This qualitative ethics study was inspired by the rare opportunity the SQUEEZE pilot trial presents to generate new knowledge on the experiences of parents/guardians with the exception to consent process. RCTs involving use of an exception to consent process are uncommon and even more so when the population under study is children. As principal investigator for the SQUEEZE pilot trial, MJP identified a responsibility to use this opportunity to advance our understanding of SDM experiences with research of this nature and contacted LS to seek out the possibility of a collaboration.

This resulting study supports creation of a ‘virtuous learning loop’,^60^ whereby the qualitative findings will inform not only study procedures in the planned multicentre RCT but also contribute to development of evidence-based guidelines that may be used by other investigators who wish to conduct research that requires use of an exception to consent process. It is exciting to see new evidence emerging in this area from other groups as well.^22^ ^23^ ^61^ Evidence-based ethics guidelines will assist investigators in developing study operating procedures for approaching SDMs to ensure that this occurs in a manner consistent with SDM preferences. The medical community has embraced the concept of ‘patient-centred care’, and perhaps it is time for the research community to consider embracing what could be referred to as ‘SDM-centred ethics’. The concept of SDM-centred ethics would require an acknowledgement that what SDMs want (when acting in the best interests of an incapable individual) may not be the same as what REBs require (based on current research ethics policies), or what ethicist may consider most virtuous. Moving towards an evidence-based approach to research participation may result in an improved research experience for SDMs and promote retention of participants in such studies. This is a much needed area of study highlighted by a call for public input on the TCPS section on use of an exception to consent.^62^

From a methodological perspective, we acknowledge that our qualitative data will be based on parent/guardian recall and accept this as an inherent limitation of our study design. While other investigators have successfully audio recorded informed consent discussions in real time,^63^ we believe that it would be impracticable to attempt similar methods in our study where the RCT consent process is typically occurring in the intensive care unit (ICU) environment and may involve multiple interactions between the CRA and SDM(s). Moreover, we do not wish to burden parents/guardians with concurrent requests for consent, which may risk not only coercion but also participant retention in the parent trial. To assess the reliability of our qualitative data, study participants will be offered the opportunity to participate in a second interview during which the data from the first interview will be reviewed, discussed and revised as needed. Strengths of our protocol include an intentional structuring of study procedures to mitigate real or perceived competing interests of the PI of the parent trial and incorporation of a novel safety plan for research staff to follow should they witness emotional distress in a research participant. The latter is particularly relevant given recent findings of high levels of depression among caregivers of ICU survivors.^64^ Our protocol has also been designed to meet recommended reporting requirements for qualitative research.^65^ ^66^

In summary, this qualitative study is the product of an innovative collaboration between the principal investigator of an RCT employing an exception to consent process and a Bioethicist. Collaborations of this nature require mutual trust, a commitment to sharing perspectives and a willingness of the clinical researcher to expose themselves and the parent trial in which they are invested to scrutiny. We look forward to the findings our study will generate being used to support development of evidence-based ethics guidelines and to optimise the experiences of SDMs in future research.
