# Supplementary material for: Exploring the experiences of substitute decision-makers with an exception to consent in a paediatric resuscitation randomised controlled trial: study protocol for a qualitative research study
Source: BMJ Open. 2016 Sep 13;6(9):e012931. doi: 10.1136/bmjopen-2016-012931 (PMC5030536; doi:10.1136/bmjopen-2016-012931)
Supplement: Supplementary File 1: Telephone Contact Transcript: Invitation to Participate in Study [file bmjopen-2016-012931supp1.pdf]

Telephone Contact Transcript: Invitation to Participate in Study

1. Hello. Could I speak with...[name of potential participant]?

If yes: *continue to 2.*
If no: "What would be the best time to reach [potential participant]? Thank you very much."

2. My name is [research coordinator/assistant]. I am calling from McMaster University to invite you to participate in an interview-based research study. The study refers to your experiences of your child's recent enrollment into a pilot clinical trial related to his/her recent illness with septic shock (resulting from a severe infection) at the McMaster Children's Hospital. Is this a good time to talk?

If yes: *continue to 3*.
If no: "What would be the best time for me to call back? Thank you very much."

3. You are being invited to talk about your experiences of your child recently having been enrolled in a clinical trial without your prior knowledge. The researchers anticipate that with your participation, you may help other people who might experience being consented after the fact. The goal to improve the experience of participating in clinical trials, and -- more broadly -- to improve medical care. As you may recall, the name of the study that you were asked to consent to *after your child had already been entered into the study* is entitled: Pilot Study for the SQUEEZE Trial: A trial assessing whether septic shock reversal is quicker in pediatric patients randomized to an early goal directed fluid sparing strategy vs. usual care (SQUEEZE).'

If you volunteer to participate in this study, we will ask you to take part in an interview with a researcher experienced in sensitive issues. The interview will last approximately 60-120 minutes.
The interview will be conducted, when possible, in person. The interview will be conducted at your preferred location; this can be in a quiet room at McMaster Children's Hospital, McMaster University, your home, or any other private and quiet location. If an in-person interview cannot be arranged, interviews will be conducted over the telephone or Internet communication method (e.g., Skype). You will receive a $25 Chapters Gift Card as a token of appreciation for the time taken to participate in the study, and you will be reimbursed for any receipted study-related costs incurred (e.g., parking).

By all means take your time in making your decision. Feel free to discuss it with your friends and family, the investigators, any of the research team members, or your health professionals. This invitation can also be sent to you though the mail or email if you would like to have a document to refer to as you make your decision.

Please note that your responses, identifying information, and other names mentioned would be kelp confidential and anonymous from the transcripts that will be made of the interview. Your healthcare providers will not see your specific responses. Only the major

lines of thought that emerge from the interviews will be used to describe important ideas that come out of the interviews.

Does this sound like something you would like to participate in? Can I provide you with more information that can be of help to you in your decision?

If yes: *Arrange a time. Inform them that they will receive a reminder call 24hrs before their scheduled interview.* [Provide contact information in case they have to reschedule.] Thank you very much for your time.

If no: "Is it the nature of the study or the timing that is leading you to answer no?"
If 'timing' is the response: "Would you reconsider if we were to contact you again in say two-weeks time?"

Thank you very much for your time.
