# Supplementary material for: Exploring the experiences of substitute decision-makers with an exception to consent in a paediatric resuscitation randomised controlled trial: study protocol for a qualitative research study
Source: BMJ Open. 2016 Sep 13;6(9):e012931. doi: 10.1136/bmjopen-2016-012931 (PMC5030536; doi:10.1136/bmjopen-2016-012931)
Supplement: Supplementary File 2: Letter mail Contact Transcript: Invitation to Participate in Study [file bmjopen-2016-012931supp2.pdf]

Letter mail Contact Transcript: Invitation to Participate in Study

DATE McMaster University Letterhead

Subject: *Invitation to participate in an interview-based research study relating to your experiences of your child being enrolled in a pilot clinical trial without your prior knowledge.*

Dear [name(s) of potential participant(s)],

You are being invited to participate in an interview-based research study led by Lisa Schwartz (PhD) at McMaster University. The study is about your experiences as a parent/substitute decision maker. It refers to your child's enrollment into a pilot clinical trial related to your child's recent illness with septic shock (resulting from a severe infection) at the McMaster Children's Hospital.

The research team is seeking your comments and perspectives on you child having been enrolled into a pilot clinical trial without your prior consent. The researchers anticipate that with your participation, you may help other people experiencing being consented after the fact. Their goal is that in you voluntarily sharing your experiences with the researchers, improvements can be made in these experiences of participating in clinical trials, and -- more broadly -- in medical care.

As you may recall, the name of the study that you were asked to consent to *after your child had already been entered into the study* is entitled: Pilot Study for the SQUEEZE Trial: 'A trial assessing whether septic shock reversal is quicker in pediatric patients randomized to an early goal directed fluid sparing strategy vs. usual care (SQUEEZE).'

The name of the interview-based study you are here being invited to participate in is: 'Exception to Consent in Pediatric Resuscitation Research: Exploring the Experiences of Substitute Decision Makers (parents/guardians).'

In order to decide whether or not you want to be a part of the current research study, you should understand that if you volunteer, you will be asked to take part in an interview with a researcher experienced in sensitive issues. The interview will last approximately 60-120 minutes. The interview will be conducted, when possible, in person. The interview will be conducted at your preferred location; this can be in a quiet room at McMaster Children's Hospital, McMaster University, your home, or any other private and quiet location. If an in-person interview cannot be arranged, interviews will be conducted over the telephone or Internet communication method (e.g., Skype). You will receive a $25 Chapters Gift Card as a token of appreciation for the time taken to participate in the study. You will also be reimbursed for any receipted study-related costs incurred (e.g., parking).

Please note that your responses, identifying information, and other names mentioned would be kelp confidential and anonymous from the transcripts that will be made of the interview. Your healthcare providers will not see your specific responses. Only the major lines of thought that emerge from the interviews will be used to describe important ideas that come out of the interviews.

Enclosed is sheet with detailed information about the research study. Please take your time to make your decision. Feel free to discuss it with your friends and family, the investigators, any of the research team members, or your health professionals.

If you are interested in participating, you may contact the research coordinator, Sonya de Laat, or the research team leader, Lisa Schwartz, listed below. Alternatively, you can respond to the research coordinator over the phone. She will follow-up this letter with a phone call within two weeks.

Thank you in advance for your time and consideration.

Sincerely,

Lisa Schwartz, PhD

General Contact Information

Dr Lisa Schwartz
Arnold L. Johnson Chair in Health Care Ethics, McMaster University
905-525-9140 ext 22987; schwar@mcmaster.ca

Sonya de Laat,
Research Coordinator, McMaster University
905-525-9140 ext 28604
delaat@mcmaster.ca

This study has been reviewed and approved by the Hamilton Integrated Research Ethics Board.
