# Supplementary material for: Exploring the experiences of substitute decision-makers with an exception to consent in a paediatric resuscitation randomised controlled trial: study protocol for a qualitative research study
Source: BMJ Open. 2016 Sep 13;6(9):e012931. doi: 10.1136/bmjopen-2016-012931 (PMC5030536; doi:10.1136/bmjopen-2016-012931)
Supplement: Supplementary File 3: Qualitative Interview Guide for Study [file bmjopen-2016-012931supp3.pdf]

Qualitative Interview Guide for Study:
Exception to Consent in Pediatric Resuscitation Research: Exploring the Experiences of Substitute Decision Makers

Total participant time required: 60-120 minutes
Break: As many as necessary

Pre-interview briefing

The goal of this interview is to explore the experience of parents/substitute decision makers with the 'exception to consent' process in the context of a pilot randomized controlled trial comparing two resuscitation strategies for pediatric septic shock. We are interested to learn:

- What is the experience for parents/guardians asked for consent for their child to remain in a study/randomized clinical trial after the main intervention (medical care) has taken place?
- Why do parents/guardians choose to continue or withdraw their child from ongoing participation?
- What strategies, responses, resources etc. employed by the researchers to recruit families did SDMs feel helped them make their decisions? What could have been improved?

We would like to learn from your experiences in being involved in a research study without prior consent - or as is described in the medical research world: 'exception to consent'. What is important is to get a description of your experience and your perspectives about being involved in a research study without prior notification. Your responses, identifying information, and other names mentioned will be kelp confidential and anonymous. Only the major lines of thought that emerge from the interviews will be used to identify important concepts.

Please feel free to look over the consent form and ask any questions that you may have about the process.

<u>General guide for leading the interview</u>

1. Consent

- Before the interview begins conduct the informed consent process.

2. Introduction (10 minutes)

- Welcome participant(s) and introduce myself;
- Explain the general purpose of the interview;
- Explain the presence and purpose of recording equipment and give the option to opt-out of recording interview;
- Outline general ground rules and interview guidelines such as participants can end the interview at any time they want or exercise their right to refuse to answer any question(s);

- Review break schedule and where the restrooms are (if time goes beyond 1.5hrs, provide a scheduled break);
- Address the issue of privacy and confidentiality; assure participants that their participation (or withdrawal) will in no way effect the quality of care.
- Inform the participant that information discussed is going to be analyzed as a whole and that participants' names will/will not be used in any analysis of the discussion;
- Read a protocol summary to the participants.

3. Interview Guidelines

This interview will consist of structured questions. During the interview I may ask you additional questions to further clarify or elaborate your answer. You may choose not to answer a particular question, in that event please feel free to inform me. You may also ask to take a break or end the interview at any time.

Your answers and any information identifying you as a participant of this research will be kept confidential.

I would like to record this interview for data analysis and to ensure that the responses were captured and transcribed accurately. No one outside of this room, other than the transcriptionist who has signed a confidentiality agreement, will have access to these tapes and they will be stored in a password protected file on a password protected computer in a locked office and stored securely at McMaster University for 10 years. Transcripts, with all identifying information removed, will be archived and made available to other researchers via Scholars Portal Dataserve network, though McMaster University.

Do you have any questions for me, before we begin?

Main research questions (50-80 minutes; *Probes in Italics*)

First of all, I want to ask you how your child is doing now. OR I want to express my deepest condolences on the loss of your child.
With all that you have been (and are) going through, we really appreciate your agreeing to participate in this study.

1. I'd like to begin by asking you to relate to me your family/child's story leading up to and following resuscitation.

- *underlying illness?*
- *describe how you perceive the relationship of the underlying illness to the need for resuscitation*
- *has anything like this happened before?*
- *what happened differently?*

2. If not asked conversationally in during response to question 1: Has/had your child been living with an underlying or long-term illness? What is your perceived relationship of that illness with your child's ultimately needing resuscitation?

3. How did you learn about the study, and that your child was enrolled in it?
- *at what point in your child's care did you learn about the study?*
- *describe to me how the study was described to you*
- *how informed did you feel after it being explained to you?*
- *how comfortable did you feel in asking for further clarification?*
- *did you get all the information you wanted?*
- *relate to me, if you can recall, your state of mind or emotional context at the time you were invited to continue participation in the study*
- *were you able to distinguish between the various studies you were being invited to participate in? Including this qualitative study...*

4. Explain the clinical trial study to me in your own words.
- *interpretation of randomization.*
- *Do you recall what arm of the study [what medical treatment option] your child was placed in?*
- *How did this experience make you feel?*

5. Why did you elect to [continue] or [withdraw] from the study?
- *where do you think that rationale came from?*
- *ethical frame of reference (personal, community, professional values)*
- *social/community network*

6. Now that you've had a bit of time to reflect, how do you think the process of consent-after-the-fact could be improved?
- *particularly the exception to consent component,*
- *and the invitation to continue participation.*
- *should conversations have taken place prior to intervention, but when emergency arises?*
- *long before emergency, at point when it is still a theoretical possibility?*

6. You were also asked to consent to be part of other related studies (PERSEVERE, SQUEEZE). What did you think of these additional requests?
- *did you feel you had enough information to understand what the study entailed and what would be required for participation?*
- *did you feel you could decline some, none, all?*
- *what did you think of being asked to include biological specimens, but not the need to collect more samples, for the study?*
- *improvements to the consent form? Website for additional information?*

Debriefing (10 minutes)
Is there anything else that you would like to say about anything discussed today?

Thank you very much for your time. At any point if you would like to revisit your participation in this study, do not hesitate to contact us. Just to confirm, you have agreed/declined (depending on decision made while reviewing this option in the consent form) that you may be re-contacted at a later date to clarify or further explore some of the responses you provided here today. We have learned a lot from your story and appreciate gaining your perspective on these topics.
