# Supplementary material for: Exploring the experiences of substitute decision-makers with an exception to consent in a paediatric resuscitation randomised controlled trial: study protocol for a qualitative research study
Source: BMJ Open. 2016 Sep 13;6(9):e012931. doi: 10.1136/bmjopen-2016-012931 (PMC5030536; doi:10.1136/bmjopen-2016-012931)
Supplement: Supplementary File 4: Data the PI of the SQUEEZE Pilot Trial requests to share with members of the linked Qualitative Ethics Study Research Team [file bmjopen-2016-012931supp4.pdf]

Data the Principal Investigator of the SQUEEZE Pilot Trial requests to share with members of the linked Qualitative Ethics Study Research Team

Data Item	Support for Collection as part of SQUEEZE	Information Source	Shared as	Rationale
SQUEEZE Pilot Trial Participant Age at Enrolment	Approved for Collection for SQUEEZE by REB	SQUEEZE REDCap Database	Aggregate, Anonymized	Characterize sub-population of children corresponding to the parents/guardians interviewed for the Ethics Study
SQUEEZE Pilot Trial Participant Sex (M/F)	Approved for Collection for SQUEEZE by REB	SQUEEZE REDCap Database	Aggregate, Anonymized	Characterize sub-population of children corresponding to the parents/guardians interviewed for the Ethics Study
SQUEEZE Pilot Trial Participant Prior Medical Co-morbid illness (categorical data)	Approved for Collection for SQUEEZE by REB	SQUEEZE REDCap Database	Aggregate, Anonymized	Characterize sub-population of children corresponding to the parents/guardians interviewed for the Ethics Study
SQUEEZE Trial Participant Enrolment Date and Time	Approved for Collection for SQUEEZE by REB	SQUEEZE REDCap Database	Aggregate, Anonymized	Characterize Flow of the Exception to Consent Process for the sub-population of children whose parents/guardians participate in the Ethics Study
Date and Time Notification of Enrolment Document Provided to SDMs	Approved for Collection by REB	SQUEEZE REDCap Database	Aggregate, Anonymized	Characterize Flow of the Exception to Consent Process for the sub-population of children whose parents/guardians participate in the Ethics Study
SQUEEZE Records describing decision making regarding timing of approach for Full Informed Consent	TCPS2 Recommended Supporting Documentation	Secure Word Document accessible only by SQUEEZE Research Team	Participant Level Data, but not SQUEEZE Outcome Data; Anonymized	Characterize Research Team Decision Making with respect to consent approach for the sub-population of children whose parents/guardians

				participate in the Ethics Study
Date and Time SDMs Approached for full Informed Consent	TCPS2 Recommended Supporting Documentation	Secure Word Document accessible only by SQUEEZE Research Team	Aggregate, Anonymized	Characterize Flow of the Exception to Consent Process for the sub-population of children whose parents/guardians participate in the Ethics Study
Date and Time Consent Obtained/Refused for Continued SQUEEZE Participation	TCPS2 Recommended Supporting Documentation	SQUEEZE Participant Consent Form, REDCap Database	Aggregate, Anonymized	Characterize time to Full Informed Consent/Refusal for the sub-population of children whose parents/guardians participate in the Ethics Study
SQUEEZE Trial Participant PELOD2 Score (continuous score; share highest recorded value)	Approved for Collection by REB	SQUEEZE REDCap Database	Aggregate, Anonymized	Characterize the sub-population of children whose parents/guardians participate in the Ethics Study. The PELOD2 score (Pediatric Logistic Organ Dysfunction Score) is a measure of organ dysfunction and therefore illness severity
SQUEEZE Trial Participant highest Vasoactive Medication Score (continuous score; share highest recorded value)	Approved for Collection for SQUEEZE by REB	SQUEEZE REDCap Database	Aggregate, Anonymized	Characterize the sub-population of children whose parents/guardians participate in the Ethics Study. The Vasoactive Medication Score is based on the amount of medication required to support the blood pressure for children in shock i.e. a measure of how severe their septic shock was from a hemodynamic perspective.

SQUEEZE Trial Participant Received Invasive or Non-invasive Mechanical Ventilation during the Intervention Phase (Y/N)	Approved for Collection for SQUEEZE by REB	SQUEEZE REDCap Database	Aggregate, Anonymized	Characterize the sub-population of children whose parents/guardians participate in the Ethics Study
SQUEEZE Trial Participant Received any form of dialysis during the Intervention Phase (Y/N)	Approved for Collection for SQUEEZE by REB	SQUEEZE REDCap Database	Aggregate, Anonymized	Characterize the sub-population of children whose parents/guardians participate in the Ethics Study
SQUEEZE Trial Participant Received any blood products in transfusion during the Intervention Phase (Y/N)	Approved for Collection for SQUEEZE by REB	SQUEEZE REDCap Database	Aggregate, Anonymized	Characterize the sub-population of children whose parents/guardians participate in the Ethics Study
SQUEEZE Trial Participant Mortality status (90 day)	Approved for Collection for SQUEEZE by REB	SQUEEZE REDCap Database	Aggregate, Anonymized	Characterize the sub-population of children whose parents/guardians participate in the Ethics Study
First and Last Name of Parent/Guardian of SQUEEZE Trial Participant	Required Information included on REB-approved SQUEEZE Consent Form	SQUEEZE Trial Consent form	Participant-level data; Not SQUEEZE Outcome Data	Required for Qualitative Ethics Study Approach for Consent
Contact telephone number(s) – Primary, secondary	Not applicable	Medical Record of SQUEEZE Participant	Participant-level; Not SQUEEZE Outcome Data	May be Required for Qualitative Ethics Study Approach for Consent.
Mailing address if provided for Parent/Guardian of SQUEEZE Trial Participant	Not applicable	Medical Record of SQUEEZE Participant	Participant-level; Not SQUEEZE Outcome Data	May be Required for Qualitative Ethics Study Approach for Consent.
Disposition of SQUEEZE Trial Participant (In hospital – if so,	Known to SQUEEZE Research Team, as this information is	SQUEEZE Research Assistant +/- REDCap	Participant-level; Participant Location, if	Required for Qualitative Ethics Study Approach for Consent

provide location; Discharged)	required to follow-up the SQUEEZE participant according to the approved protocol.	Database	in hospital, is not SQUEEZE Outcome Data	
Mortality status as last documented in the REDCap database or otherwise known to the SQUEEZE Research Assistant at the time the Parent/Guardian of the SQUEEZE Participant is to be approached by the Qualitative Research Assistant	Known to SQUEEZE Research Team; mortality status is tracked as an approved study outcome.	SQUEEZE Research Assistant +/- REDCap Database	Participant-level data; SQUEEZE Outcome Data	Advisable for this information to be disclosed to the Qualitative Research Assistant who will be contacting the parent/guardian to know whether parents they are contacting are bereaved.
