# Supplementary material for: Exploring the experiences of substitute decision-makers with an exception to consent in a paediatric resuscitation randomised controlled trial: study protocol for a qualitative research study
Source: BMJ Open. 2016 Sep 13;6(9):e012931. doi: 10.1136/bmjopen-2016-012931 (PMC5030536; doi:10.1136/bmjopen-2016-012931)
Supplement: Supplementary File 5: Procedures for Responding to Obvious Emotional Distress in a Qualitative Research Participant [file bmjopen-2016-012931supp5.pdf]

**Procedures for Responding to Obvious Emotional Distress in a Qualitative Research Participant**

Given the nature of this research, we anticipate that the QRA may directly observe obvious emotional distress in research participants during or at completion of the interview process. Under such circumstances, and depending on the perceived level of distress, one of the following actions will be taken by the QRA and documented based on their best judgment of the situation, recognizing that they are not a mental health professional.

1. Where the QRA notes signs of emotional distress (participant verbally expresses and/or is visibly upset and/or is tearful) at the interview, they will offer information about psychosocial support for parents of sick children and where applicable for bereaved parents. They will also advise the participant to follow up with their family physician or other primary healthcare professional to further explore their feelings and/or the need for counseling.

2. Where the QRA feels that there is an <u>urgent</u> need for evaluation of the participant's emotional state that should not wait for assessment by a primary health care provider, they will advise the participant to go to the Emergency Department for assessment. Emergency Physicians are trained and qualified to evaluate risk in patients presenting with acute mental health problems, and they can facilitate acute psychiatric evaluation if this is determined to be clinically warranted.

3. Where the QRA believes that the participant is at <u>imminent risk</u> of harm to himself or herself or someone else i.e. the participant expresses acute suicidality or homicidality, the QRA will call (telephone) 911. Calling 911 activates a variety of emergency response

services including the police and ambulance services. The police service is capable of locating and transporting individuals with acute mental health problems to the Emergency Department for assessment. Individuals may also be transported by ambulance, with or without police accompaniment.
